# The Impact of ASCITIC Paracentesis on Symptomatic Outcomes in Palliative Patients: 3-Year Experience of a Melbourne Inpatient Palliative Care Unit Using Palliative Care Outcomes Collaboration Assessments

**DOI:** 10.1155/2022/7300110

**Published:** 2022-10-13

**Authors:** Kiran Iyer, Lucy Kernick

**Affiliations:** ^1^St Vincent's Hospital, Melbourne, Australia; ^2^Mercy Health, Melbourne, Australia

## Abstract

**Background:**

Ascites formation is a common occurrence in patients receiving palliative care. This is associated with symptoms that may respond to drainage.

**Aim:**

To review and quantify ascites-related symptoms pre- and post-paracentesis to evaluate its benefit in an inpatient palliative care setting.

**Methods:**

A retrospective audit of patients admitted to an inpatient palliative care unit who underwent paracentesis between November 2016 to June 2020 was performed. The primary outcome was a symptomatic benefit of paracentesis. Secondary outcomes assessed the associated complications as well as the alterations to functional status. Data were analysed using a paired *T*-test.

**Results:**

43 instances of ascitic paracentesis were performed on a total of 26 patients. Three patients were excluded from the study due to the technical failure of their paracentesis procedure. There was a mean 0.16-point reduction in pain (95% confidence interval (CI) −0.33 to 0.65), a mean 0.03-point increase in dyspnoea (95% CI −0.51 to 0.46), and a mean 0.32-point reduction in nausea (95% CI −0.09 to 0.74).

**Conclusions:**

Ascitic paracentesis in the palliative setting may demonstrate some benefit in managing symptoms associated with ascites. Although the findings of this study did not achieve statistical significance, these results may be substantiated by future studies with larger sample size.

## 1. Background

The formation of ascites is a common occurrence in palliative care patients and may be malignant or non-malignant in origin [1]. Management options can include medical management by diuresis, serial paracentesis, transjugular intrahepatic portosystemic shunts, or tunnelled intra-abdominal catheterisation [2].

In a palliative care setting, the management goals are directed at symptom control and improvement in quality of life [3]. The symptoms produced by ascitic fluid accumulation can include abdominal pain, dyspnoea, nausea, and abdominal distension; symptoms that may be mitigated with procedural intervention [4]. Current recommendations suggest palliative drainage of ascitic fluid, with a reported symptomatic benefit of 64–100% of patients in a systematic review of 32 original articles [5]. This wide range of reported symptomatic benefits may reflect varying patient populations, techniques, and subjective symptomatic data.

## 2. Aim

To review and quantify symptoms pre- and post-paracentesis in those who underwent the procedure during admission to an inpatient palliative care unit, by using the Palliative Care Outcomes Collaboration (PCOC) assessments.

## 3. Methods

We conducted a retrospective audit of all ascitic paracentesis procedures performed for patients admitted to a single palliative care centre over a three-year period from 1 November 2016 to 1 June 2020. Ethics approval was obtained by the institutional ethics committee, with research conducted in accordance with the National Statement on Ethics Conduct in Human Research (2007). The relevant patients were identified through extraction of ascitic paracentesis codes, excluding patients not admitted to the palliative care unit. These data were cross-referenced with existing medical records. Data were obtained from a medical record review. This included information from progress notes, pathology records, fluid balance forms, and the PCOC Symptom Assessment Scale. It should be noted that the symptom assessment scale attempts to measure the distress a patient ascribes to the symptom, rather than the intensity of the symptom itself.

This study did not distinguish between malignant and non-malignant ascites when assessing the symptomatic benefit of ascitic paracentesis. The primary outcome was a symptomatic benefit, defined as the difference in PCOC symptom assessment score pre-paracentesis versus post-paracentesis. This attempts to identify any changes in the patient's distress level related to certain symptoms pre- and post-intervention. Data were analysed using a paired *T*-test, comparing patient symptom scores prior to and after paracentesis. Secondary outcomes include the rate of post-paracentesis complication and mortality post-paracentesis.

## 4. Results

Overall, a total of 43 ascitic paracentesis procedures were performed on 26 patients in the three-year time period. An average of 1.65 procedures were performed per patient, with a range of 1–6 procedures. These patients were followed from their first presentation until their death. Characteristics of patients are summarised in Table 1. This study group contained 13 males and 13 females (Age range = 40–89, Mean = 68.65). 20 patients (76.96%) were documented to have malignant ascites versus three patients (11.54%) who had non-malignant ascites and a further three patients (11.54%) had ascites of a mixed aetiology. Common malignant aetiologies included pancreatic (21.74%), cholangiocarcinoma (21.74%), colorectal (17.39%), and breast (13.04%). Other malignant causes included endometrial (8.70%), hepatocellular (8.70%), gastric (4.35%), and oesophageal malignancies (4.35%).

Details of interventions and complications are summarised in Table 2. Abdominal pain (53.85%) and abdominal distension (46.15%) represented the largest symptomatic indications for ascitic paracentesis. Other symptomatic indications included nausea (38.46%), dyspnoea (34.62%), anorexia (7.69%), and gastro-oesophageal reflux (3.85%). An average volume of 6.01 L (range = 0.6–17.15 L) of ascitic fluid was drained per intervention, albumin was administered in 27.91% of cases, and 1 or more complications occurred in 34.88% of interventions. Complications of ascitic paracentesis occurred in 28 (65.12%) cases. These included 6 cases of hypotension, 5 cases of drain dislodgement, 3 cases of haemorrhage, and 1 case of worsening pain.

In the analysis of the pre- and post-paracentesis symptom assessment scores as seen in Table 3, there was a 0.16-point reduction in the mean pain score (95% CI −0.33 to 0.65), and there was a 0.32-point reduction in the mean nausea score (95% CI −0.09 to 0.74). Scores for abdominal distension were not analysed as this was not measured by the PCOC Symptom Assessment Scale. Of the 26 patients, 18 died during admission when they underwent ascitic paracentesis. A further 4 patients died in later admissions, 3 died in the community setting and 1 patient was lost to follow-up. The mean time to death from paracentesis was 17.92 days, with a median time of 11 days.

## 5. Discussion

Currently, the symptomatic management of ascites in palliative patients is through serial paracentesis, with the aim of draining abdominal ascites to ease the associated symptoms [5]. Within this study, both abdominal pain and nausea had a reduction in their post-paracentesis symptom score. Conversely, the opposite was true of dyspnoea, with a minor increase in post-paracentesis symptom score. However, the results were neither statistically nor clinically significant.

Confounding variables to be considered include the volume of ascites drained, the impact of intravenous albumin administration, and the impact of paracentesis-related complications on symptom scores. In this study, the volume of ascites drained ranged from 0.6 up to 17.15 L per intervention. As a standard volume was not drained per patient, it is unclear what effect the varying volumes had on the post-paracentesis symptom scores. Furthermore, the administration of intravenous albumin was not standardised across all patients, which may have had an impact on the development of post-paracentesis complications. This lack of standardisation may be reflected in varying practitioner preferences, lack of local standardised protocol, and a paucity of supportive evidence for intravenous albumin administration in malignant ascites management [6, 7]. This lack of standardisation of ascitic paracentesis protocol highlights a key limitation within this study. Given differing aetiologies and physiology, the origin of the ascites may impact the management – and therefore symptoms. Additionally, this study does not analyse and delineate between malignant and non-malignant ascites. Within our limited data set, there was no association between albumin administration and a reduction in hypotensive episodes.

The key strength of this study lays in its standardised methodology. Through the usage of the PCOC symptom assessment scale, qualitative data such as pain, dyspnoea, and nausea, and translated into qualitative data for analysis. Due to the standardised formatting and protocol, this data could be collected by nursing staff with reduced inter-patient variability across palliative care settings. Additionally, patients were compared against themselves in assessing the symptomatic benefit of paracentesis intervention. As patients have subjective experiences and interpretations of their symptomatology [8], patients were compared against themselves instead of a separate placebo group. Hence, instead of trying to match participants on the basis of similar characteristics, this study highlights the trend in patient symptom scores for each patient.

However, there are several limitations to this study. This was a low-powered study, and as a result, there were significant implications for the statistical analysis. The small sample size was reflected in the wide range of confidence intervals. The symptom of pain was only assessed by one method. The symptom assessment score describes “pain” as the symptom, yet almost half the patients in this study were referred for abdominal distension. The tool used may have missed this symptomatology and, hence, under-estimated the symptom burden. Furthermore, given that this study treated pre-paracentesis data as the control group, and post-paracentesis data as the intervention group, this study fails to account for the placebo effect. To account for the placebo effect, blinding the patient to exposure would be required. This would have proven difficult with regards to methodology and ethical considerations.

The documented benefits seen in this sample were less than expected by the treating team. Whilst there were limitations to this study, it raises the question of whether the procedure is as effective for symptom management as thought. Further studies are required incorporating a larger sample size and broader symptom assessment tools.

## 6. Conclusions

This study showed that in those patients who underwent ascitic paracentesis for palliative management of ascites, there was no statistically significant difference in symptoms. This study addresses the weakness of the previous study here the assessment of symptomatic benefit post ascitic paracentesis is largely based on subjective data. Future studies may be improved with standardised ascitic paracentesis protocol, larger sample sizes, and a delineation between malignant and non-malignant ascites groups.

## Figures and Tables

**Table 1:** Characteristics of patients.

	*n* = 26
Age (years), range	68.65, 40–89
Male gender *n*, (%)	13 (50%)
Female gender *n*, (%)	13 (50%)
Admissions per person median, (range)	1(1–6)
Length of stay *n* (days), (range)	15.54, (1–82)
Aetiology of ascites *n*, (%)	
Malignant	20 (76.92%)
Non-malignant	3 (11.54%)
Mixed	3 (11.54%)
Aetiology of malignant ascites *n*, (%)	
Pancreatic cancer	5 (21.74%)
Cholangiocarcinoma	5 (21.74%)
Colorectal cancer	4 (17.39%)
Breast cancer	3 (13.04%)
Endometrial cancer	2 (8.70%)
Hepatocellular cancer	2 (8.70%)
Gastric cancer	1 (4.35%)
Oesophageal cancer	1 (4.35%)

**Table 2:** Details of interventions and complications.

	*n* = 48
Symptom indication for Ascitic paracentesis n,	
(% of cases with this symptom as an indication)	
Abdominal distension	14 (53.85%)
Abdominal pain	12 (46.15%)
Nausea	10 (38.46%)
Dyspnoea	9 (34.62%)
Anorexia	2 (7.69%)
Reflux	1 (3.85%)
Number of Ascitic paracentesis interventions per patient mean, (range)	1.65 (1–6)
Complications	
Present *n*, (%)	15 (34.88%)
Hypotension *n*, (%)	6 (40%)
Dislodged tap *n*, (%)	5 (33.33%)
Bleeding *n*, (%)	3 (20%)
Pain *n*, (%)	1 (6.66%)
Absent *n*, (%)	28 (65.12%)

**Table 3:** PCOC SAS.

	Pre-paracentesis	Post-paracentesis
Pain		
Mean	1.79	1.53
Median	0	0.5
Range	0–7	0–7
Dyspnoea		
Mean	0.79	0.78
Median	0	0
Range	0–7	0–7
Nausea		
Mean	0.47	0.3
Median	0	0
Range	0–4	0–4

## Data Availability

The observational data used to support the findings of this study are available from the corresponding author upon request. Kindly email the author at kiyer1996@gmail.com to request for the data used in this study.
